# Impact of Tailored-Enhanced Recovery After Surgery Versus Conventional Care in Patients of Gastro-Duodenal Perforation: A Pilot Randomized Control Trial

**DOI:** 10.7759/cureus.45349

**Published:** 2023-09-16

**Authors:** Cherring Tandup, Abhinav Chauhan, Rajeev Chauhan, Vipul Thakur, Swapnesh Sahu, Lileswar Kaman, Siddhant Khare, Yashwant Sakaray, Krishna N Nenavath, Kailash C Kurdia

**Affiliations:** 1 General Surgery, Postgraduate Institute of Medical Education and Research, Chandigarh, Chandigarh, IND; 2 Anaesthesia and Intensive Care, Postgraduate Institute of Medical Education and Research, Chandigarh, Chandigarh, IND

**Keywords:** surgical site infection(ssi), gastro-duodenal perforation, emergency, length of hospitalization, enhanced recovery after surgery

## Abstract

Background: The enhanced recovery after surgery (ERAS) program established improved clinical outcomes in elective surgery; however, its role in emergencies is uncertain. This study was designed to assess the feasibility, safety, and efficacy of a tailored-ERAS (t-ERAS) protocol in patients undergoing modified Graham’s patch closure for gastro-duodenal perforation.

Methods: A single-centre, prospective, parallel-arm, open-label, randomized controlled trial was conducted from February 2021 to December 2021. Patients with gastroduodenal perforation undergoing modified Graham's patch were randomly assigned to either conventional care or the t-ERAS pathway. Patients with refractory septic shock, psychiatric or neurological disorders, pregnancy, multiple perforations, sealed-off perforations, and perforation sizes greater than 1.5 cm were excluded. The primary outcome was to compare the length of hospitalization (LOH). Functional recovery parameters and morbidity were compared in secondary outcomes.

Results: Twenty-five patients each were included in conventional care and the t-ERAS group. In the t-ERAS group, LOH was significantly shorter (6.3 SD2.15 days versus 9.56 SD4.33 days, p = 0.001). Patients in the t-ERAS group had significantly early functional recovery (days) with time to first bowel sound (1.8 SD0.41; p 0.002), first flatus (2.52 SD0.65; p = 0.026), first stool (3.04 SD0.68; p < 0.001), first liquid diet (2.24 SD0.60; p = 0.002), and duration of ileus (2.64 SD0.86; p = 0.038). There was no significant difference in morbidity such as post-operative nausea and vomiting, SSI, or pulmonary complications between the two groups.

Conclusion: Tailored ERAS pathways are safe and effective in reducing the LOH and promoting early functional recovery in patients undergoing emergency closure of gastro-duodenal perforation.

## Introduction

A perforated gastro-duodenal ulcer is one of the most common surgical emergencies, occurring as a complication of peptic ulcer disease (PUD), non-steroidal anti-inflammatory drug (NSAID) abuse, and gastric cancer [1]. Although, with effective medical therapies, there has been a dramatic decrease in the rate of elective surgical procedures for PUD, the requirement for emergency surgery for complications such as perforation and bleeding has remained the same or increased [2,3]. About 2-10% of patients with PUD present with perforation in an emergency [4]. Perforated peptic ulcer disease (PPUD) is associated with a substantial mortality rate of 6-30% and is responsible for more than 70% of deaths associated with PUD [4,5]. The management of perforation is done by simple procedures such as open primary closure or modified Graham patch repair. Laparoscopic options are now being developed to reduce morbidity [6-8]. Though promising advancements have been made in the fields of surgery, anaesthesia, and medicine, the postoperative management of PPUD is based more on old traditional practices than evidence-based ones.

Enhanced recovery after surgery (ERAS) pathways developed by Henrik Kehlet in the 1990s encompass a multimodal evidence-based approach at every step of perioperative care, including the rehabilitation phase. These have now become the standard of care across specialties, reducing hospital stays and postoperative morbidity and mortality [9]. The ERAS programme has been widely implemented in elective surgical procedures; however, perioperative care in emergency surgeries continues to use the conventional protocol of management [10]. Recent evidence has demonstrated that many conventional care principles are baseless and harmful [9].

Literature on the use of ERAS pathways in emergency settings is scarce due to challenges in applying the elements of ERAS pathways in emergencies [11]. ERAS programmes are often modified in elective procedures on an individual or institutional basis, and so they may have a role in emergency settings if adapted and tailored [12].

This randomized study aimed to investigate the feasibility, safety, and efficacy of the tailored-ERAS protocol (t-ERAS) in patients who underwent modified Graham patch closure of gastro-duodenal perforation.

## Materials and methods

This study was a single-institutional, prospective, parallel-arm, open-label, randomized controlled trial carried out in the Department of General Surgery of a tertiary care hospital in India from February 2021 to December 2021. This study was approved by the Institution Ethics Committee and registered under INT/IEC/2020/SPL-1341, and the study was also registered at www.ctri.gov.in (CTRI/2021/01/030774). A written informed consent was obtained from the participants enrolled.

All patients above the age of 18 years presenting within 48 hours of the onset of symptoms to the emergency department and diagnosed as having gastro-duodenal perforation were enrolled and assessed for eligibility. A clinical diagnosis of gastro-duodenal perforation was made by clinical history and examination, supported by radiological investigations. Patients presenting with refractory septic shock, psychiatric or neurological disorders, and pregnant women were excluded. After randomization, patients with sealed-off perforations requiring no operative intervention, patients with multiple perforations, perforation sizes greater than 1.5 cm, or patients requiring additional procedures were excluded. Only patients receiving modified Graham patch closures were included.

Randomization

Participants were randomly assigned to either conventional care or the tailored ERAS pathway in a 1:1 ratio. For randomization, an online website was used, and for allocation concealment, the Serially Numbered Opaque Sealed Envelope (SNOSE) technique was used. An individual unrelated to the investigators was preparing the opaque, sealed envelope, which was opened by a theatre staff member after the decision to proceed with surgery was made.

Preoperative care

The preoperative management was comparable in both groups. Nasogastric (NG) tube placement and urethral catheterization were done in all patients on admission. Intravenous crystalloids were administered for fluid replacement, and intravenous antibiotics, cefuroxime (1.5 mg 12 hours a day) and metronidazole (400 mg 12 hours a day), were administered after sensitivity testing. Baseline investigations such as complete blood counts (CBC), renal function and liver function tests, coagulation profiles, and arterial blood gas analysis were performed. Patients in both groups were counselled to alleviate any anxiety, fear, or pre-notions regarding surgery. Hypothermia was prevented by providing a warm environment.

Intraoperative management

In the operating room (OR), standard monitoring was performed following American Society of Anesthesiologists (ASA) guidelines, including ECG, SpO_2_, non-invasive pressure (NIBP), EtCO_2_, and temperature monitoring. In the ERAS group, we had planned to insert lumbar epidural catheters in T10-T11 interspaces in patients who had no contraindication, but due to logistical reasons, only two patients received epidural catheterization. Patients in both groups were preoxygenated with 100% oxygen, followed by induction with intravenous thiopentone (5 mg/kg) and succinylcholine (2 mg/kg) in a rapid sequence induction (RSI). Sevoflurane was used to maintain anaesthesia, with a MAC value of 1 in the conventional group and a MAC value of 0.5-0.8 in the ERAS group. Short-acting opioids were administered when necessary in the ERAS group. A midline laparotomy was performed, and intraoperative findings were recorded. A modified Graham patch repair for perforation was followed by a rigorous peritoneal lavage and subhepatic drain placement. Intraoperative temperature monitoring was ensured to prevent hypothermia.

Postoperative care

The t-ERAS group included early mobilization, nutrition, non-opioid analgesia, and early removal of abdominal drains. Patients in conventional care received opioid analgesia on days 0 and 1 of the postoperative period, followed by acetaminophen with or without opioid analgesia. In the t-ERAS group, non-opioid multi-modal analgesia was used, and opioid analgesia was used for breakthrough pain. For postoperative nausea and vomiting (PONV), metoclopramide was used. Early ambulation from postoperative day zero was practiced in the t-ERAS group. If an epidural catheter was inserted, sitting for two to three hours on the day of surgery and ambulation after removal of the epidural catheter were practiced. Early removal of the drain, urinary catheter, and NG tube was followed in t-ERAS, whereas in conventional care, the urinary catheter was removed after the third postoperative day and drains were removed after the patient tolerated oral feeds. Differences in postoperative care in both groups have been described in Table 1. Patients in both groups were discharged when they tolerated a solid diet for at least one day with no fever or infected wound. Patients in both groups were followed for 30 days post-discharge.

**Table 1:** Tailored ERAS pathway versus conventional care ERAS: enhanced recovery after surgery, IV: intravenous, NG: nasogastric, POD: postoperative day, PPI: proton pump inhibitor

Primary component	Tailored ERAS pathway	Conventional perioperative care
Preoperative management (both groups had NG tube placed, IV antibiotics, and PPIs)	Non-opioid multimodal analgesia (IV acetaminophen and lumbar epidural analgesia	Opioid analgesics (inj. Tramadol 50 mg IV 8^th^ hourly)
	Opioids for breakthrough pain (inj. Tramadol 50 mg IV)	
Intraoperative management (all patients underwent modified Graham’s patch repair under general anaesthesia)	Short acting opioids and anaesthetic agents when necessary (Fentanyl 1 microgram/kg, Sevoflurane 0.5–0.7 minimum alveolar concentration)	Standard anaesthetic protocols
	Epidural lidocaine in patients who had epidural catheter (16 ml of 1% lidocaine with 150 microgram of adrenaline)	
Postoperative management postoperative analgesia	Non-opioid multimodal analgesia POD 0-2 - Epidural local anaesthetic infusion and IV Acetaminophen 8^th^ hourly	Opioid analgesia POD 0-2 - IV tramadol
	POD 3 - oral acetaminophen 500 mg 8^th^ hourly (IV dose if oral feeds not resumed)	POD 3 onwards - IV tramadol and acetaminophen
	POD 4 - oral acetaminophen as needed	Oral doses once feeds resumed
	Breakthrough pain - opioids (based on Likert scale)	Breakthrough pain - opioids
Adjuvant medication	POD 0-1 - IV metoclopramide 10 mg 8^th^ hourly	IV PPIs → oral doses
	IV PPIs → oral doses	
Mobilization	POD 1 (If epidural catheter inserted; sitting for 2 hours on the day of surgery)	POD 2 or further till patient is comfortable and not complained of any significant pain
Withdrawal of NG tube	POD1 irrespective of presence or absence of bowel sounds	Bowel sound present and after passage of flatus/stool (POD 3-4)
Withdrawal of drains	Output ≤ 100 ml/day irrespective of resumption of oral feeds	Unrestricted liquid diet tolerated × 24 h (POD 5/6)
Withdrawal of urinary catheter	Urine output adequate over the last 24 h (1 ml/kg/h in absence of inotropes/diuretics)	POD 3
Resumption of oral feeds (if intolerance to diet, feeds stopped and restarted once asymptomatic)	POD 1 - sips	NPO till resolution of the ileus
	POD 2-3- clear water (500 ml)	Restricted volumes of clear fluid
	POD 4-soft diet	Soft diet/ normal diet as tolerated (POD 4-5)

Outcome

The primary outcome was to compare the length of hospital stay between the two groups. Our secondary outcome was to compare functional recovery, which is a compound term demanding fulfilment of several indicators of well-being such as postoperative duration of ileus, time to passage of flatus and stool, removal of NG tube, abdominal drains and catheters, time to resume oral feeds, and time to mobilize. We also compared the morbidities, such as the need for added analgesia, the incidence of PONV, surgical site infections (SSI), pulmonary complications, and urinary tract infections. Re-admission within 30 days of discharge from the hospital was assessed.

Statistical analysis

The data were recorded in a pre-designed proforma and were analyzed with the help of SPSS 19.0 (IBM Corp., Armonk, NY). Continuous variables, for example, age, perforation size, time for removal of the NG tube/drain, duration of surgery, time for resolution of the ileus, starting of oral feeding, and length of hospital stay, were tabulated as means with standard deviation. An independent student t-test, or Mann-Whitney U test, was used to analyze continuous variables. The chi-square test, or Fischer’s exact test, was used to analyze categorical variables. A p-value of less than 0.05 was considered significant.

## Results

**Figure 1:**
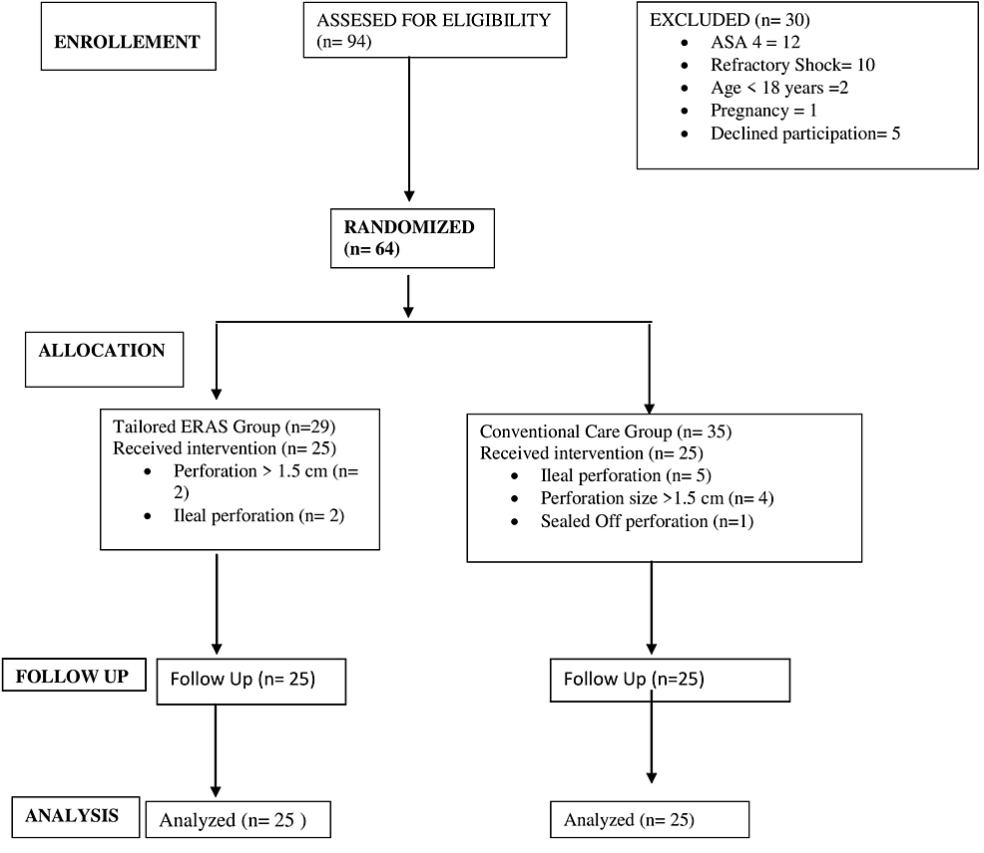
CONSORT diagram

A total of 94 patients with gastro-duodenal perforation were assessed for eligibility; 64 patients were randomized, 35 to the conventional care group, and 29 to the t-ERAS group (Figure 1). In the conventional care group, five ileal perforations, four greater than 1.5 cm perforations, and one sealed-off perforation patient were excluded. In the t-ERAS group, two ileal perforations and two perforations greater than 1.5 cm were excluded. So, a total of 50 patients were included for analysis, and no patient was lost to follow-up during the study period. The two groups were comparable in terms of demography, co-morbidities, and pre-operative characteristics, as shown in Table 2.

**Table 2:** Demographics, co-morbidities and pre-operative characteristics ERAS: enhanced recovery after surgery, SD: standard deviation, BMI: body mass index, ASA: American Society of Anesthesiologists, CKD: chronic kidney disease. *Significant at p<0.05 level.

Variable	Tailored-ERAS group (n=25)	Conventional care group (n=25)	P-value
Mean age (in years)± SD	44.28 ±12.10	43.44 ±11.82	0.54
Gender			
Male (%)	24 (96%)	23 (92%)	1.00
Female (%)	1 (4%)	2 (8%)	
BMI ± SD	23.15 ±3.38	22.14 ±3.46	0.30
ASA			
Class I	19 (76%)	10 (40%)	0.02*
Class II	6 (24%)	13 (52%)	
Class III	0 (0)	2 (8%)	
Presence of co-morbidities			
Diabetes	0 (0%)	3 (12%)	0.23
Hypertension	3 (12%)	6 (24%)	
Tuberculosis	1 (4%)	0 (0%)	
CKD	0 (0%)	1 (4%)	
None	20 (80%)	14 (56%)	
Others	1 (4%)	1 (4%)	
Duration of illness (hrs)± SD	37.44 ±18.24	50.88 ±30.24	0.06
Mean pulse rate ± SD	89.56 ±6.62	89.24 ±7.88	0.87
Blood pressure			
Systolic blood pressure (mmHg) ± SD	112.68 ±7.75	113.28 ±9.82	0.81
Diastolic blood pressure (mmHg) ± SD	76.52 ±10.77	76.68 ±9.86	0.96
Intra-op contamination			
Mild	1 (4%)	5 (20%)	0.07
Moderate	19 (76%)	20 (80%)	
Severe	5 (20%)	0 (0%)	
Nature of exudates			
Bilious	2 (8%)	2 (8%)	0.22
Purulent	1 (4%)	5 (20%)	
Bilious purulent	22 (88%)	18 (72%	

Primary outcome

The length of hospital stay was observed to be significantly reduced in the t-ERAS group in comparison to the conventional care group. The mean length of hospital stay for participants in the t-ERAS group was 6.3 SD 2.15 days, whereas it was 9.56 SD 4.33 days for participants in the conventional group. There was a statistically significant difference in length of hospital stay with a p-value of 0.001 (Table 3).

**Table 3:** Primary and secondary outcomes ERAS: enhanced recovery after surgery, CI: confidence interval, SD: standard deviation. *Significant at P<0.05 value.

Outcome variables	Tailored-ERAS group (mean) (n-25)	Conventional care group (mean) (n=25)	Mean difference	CI	P-value
Length of hospital stay (days) ± SD	6.32 ± 2.15	9.56 ± 4.33	3.24 ± 0.97	1.29–5.18	0.001*
Time to first bowel sound (days) ± SD	1.80 ± 0.41	2.28 ± 0.60	0.48 ± 0.15	0.18–0.77	0.002*
Day of nasogastric tube withdrawal (days) ± SD	1.64 ± 0.49	2.56 ± 1.23	0.92 ± 0.26	0.387–1.45	0.001*
Time to first flatus (days) ± SD	2.52 ± 0.65	3.00 ± 0.82	0.48 ± 0.21	0.05–0.90	0.03*
Time to first stool (days) ± SD	3.04 ± 0.68	4.00 ± 0.71	0.96 ± 0.20	0.56–1.35	<0.001*
Time to first liquid diet (days) ± SD	2.24 ± 0.60	3.00 ± 0.76	0.36 ± 0.23	0.1–0.82	0.002*
Time to removal of urinary catheter (days) ± SD	2.04 ± 0.20	3.12 ± 1.17	1.12 ± 0.24	0.64–1.5	<0.001*
Time of removal of drain (days) ± SD	3.20 ± 0.58	4.04 ± 0.98	0.84 ± 0.23	0.38–1.29	<0.001*
Need for extra analgesia-n (%)	2 (8.70)	6 (26.09)			0.243

Functional recovery outcome

In the secondary outcomes, participants in the t-ERAS group had significantly earlier resolution of ileus, early bowel sounds, and early removal of the nasogastric tube (Table 3) compared to patients in the conventional care group.

In terms of bowel function, patients in the t-ERAS group had a significantly early return of bowel function, with early passage of the first flatus and early passage of the first stool in the postoperative period and an early start of oral liquid followed by a solid diet.

There was a significant difference in the time of removal of the urinary catheter and intraperitoneal drain between the groups, with the patients in t-ERAS having early removal as compared to the conventional care group, and no patient in any group needed re-catherization (Table 3).

Morbidity outcome

In terms of various postoperative morbidity parameters, no significant difference was seen between the t-ERAS and conventional care groups. No significant difference in the incidence of superficial site infection, PONV, or pulmonary complications was observed (Table 4). Only two patients in t-ERAS required added analgesia in the form of opioids, whereas six patients in the conventional care group required opioid analgesia along with acetaminophen. There was no re-admission within 30 days of discharge.

**Table 4:** Comparison of postoperative complications ERAS: enhanced recovery after surgery, PONV: postoperative nausea and vomiting, SSI: surgical site infection. p-Value significant at <0.05.

	Tailored- ERAS group (n=25)	Conventional care group (n=25)	P-value
Number of patients who developed PONV	2 (8%)	5 (20%)	0.41
Superficial SSI	3 (12%)	5 (20%)	0.70
Organ space SSI with leakage	1 (4%)	3 (12%)	0.60
Pulmonary complications	2 (8%)	3 (12%)	1.00
Urinary tract infections	0 (0%)	0 (0%)	-
Re-admission (days)	0 (0%)	0(0%)	-

## Discussion

A prospective randomized controlled study was first conducted by Gonenc et al. to evaluate the feasibility of the ERAS protocol in patients with PPUD undergoing laparoscopic primary closure. The study solely focused on the postoperative ERAS care elements, which included avoiding unnecessary nasogastric decompression, resumption of early feeding, and use of non-opioid analgesia [13].

Another randomized controlled trial by Mohsina et al. compared the adapted ERAS pathway versus standard care in patients receiving simple primary closure of perforated duodenal ulcers [14]. The study demonstrated a significant reduction in hospital stay in the patients being managed by t-ERAS pathways, which was similar to our observation. In our study, there was a significant reduction in the length of hospital stay, with a mean length of hospitalization (LOH) of 6.3 ± 2.15 days in the t-ERAS group as compared to 9.56 ± 4.33 days in the conventional care group (p < 0.001). They also analyzed the functional recovery parameters and found significantly early passage of the first flatus, first stool, and early starting of a fluid diet. Their study demonstrated a significant reduction in postoperative morbidity with adapted ERAS vs. standard care [2].

There have been studies of the implication of ERAS protocols in emergency surgeries that have demonstrated a decrease in LOH, such as Roulin et al. and Lohsiriwat, who reported reduced hospital stays in patients following the ERAS protocol in cases of urgent colectomy [15,16].

Due to diffuse peritonitis in patients undergoing emergency laparotomy for perforation, intestinal motility may take up to several days to recover, so all patients are generally subjected to nasogastric decompression for a few days and kept nil per orally. So, we analyzed the functional outcomes such as the time of removal of the nasogastric tube, the time to passage of the first flatus and stool, the return of bowel movement, the initiation of the first fluid and solid diet, and the time of removal of the urinary catheter and abdominal drain. In our study, we found significantly earlier recovery in patients with t-ERAS as compared to the conventional care group in terms of the functional parameters mentioned earlier. Similar results were observed by Mohsina et al. [14].

In a study reported by Grantcharov and Kehlet, the ERAS pathway was followed in patients undergoing elective laparoscopic gastric surgery for various pathologies with the omission of placement of a nasogastric tube and starting early oral feeding, and a surgical outcome was observed [17]. They reported that ERAS pathways decreased the duration of hospital stays, while the morbidity was comparable. Gonenc et al. removed the NG tube immediately after the patient had recovered from anaesthesia; Mohsina et al. removed the NG tube when it was draining ≤300 ml over 24 hours [13,14]. In our study, the NG tube was withdrawn on postoperative day 1 in the t-ERAS group. Long-term use of NG tubes increases morbidity like prolonged ileus, increased pulmonary complications, and delay in initiating oral feeds [18]. In our study, a significantly shorter duration of ileus and early resumption of oral liquid and solid food were observed with early removal of the NG tube in the t-ERAS group. Early removal of the NG tube, early withdrawal of the drain, early oral feeding, and decreased use of opioid analgesia lead to a significantly decreased incidence of postoperative ileus.

Mohsina et al. reported a mean of 1.5 and 2.6 days for the resumption of oral liquid and solid diets, similar to the study by Gonenc et al., who reported 1.5 days in the postoperative period. Patients in the t-ERAS group started a liquid diet by 2.24 ± 0.60 days (p = 0.0002) and a solid diet by 3.16 ± 0.69 days (p = 0.017) [13,14].

Pain management is the key to achieving a successful surgical outcome following ERAS pathways. The use of multimodal analgesia by combining various analgesic agents for early recovery of patients has been practiced in ERAS pathways [2,10]. Gonenc et al. used NSAIDs for pain management, and opioids were reserved for pain not responding to NSAIDs [13]. In this study, we used intravenous acetaminophen for pain management, and opioids were reserved for breakthrough pain and administered based on the severity of pain using the Likert scale. Two patients (8.70%) in the t-ERAS group and six patients (26.09%) in the conventional care group needed extra analgesia, but it was not significant.

A significant reduction in postoperative morbidity, such as the incidence of SSIs, pulmonary complications, UTIs, and PONVs, was reported in the ERAS group [13]. A study comparing all emergency laparotomies in pre- and post-ERAS groups reported a significant decrease in postoperative complications in the post-ERAS group [19]. In patients with urgent colectomy, there was a non-significant decrease in postoperative complications in the ERAS protocol group [16]. Similarly, in our study, there was no statistically significant reduction in postoperative complications in the t-ERAS group. One patient in t-ERAS and three patients in the conventional care group had organ space infections following leaks from the repaired site, which were managed non-operatively. No readmissions within 30 days of discharge were noted in either group.

The study has some limitations. First, laparoscopic management of PPUD is implied at various centres, though we have taken an open approach due to logistical reasons. The effect of epidural analgesia on early recovery could not be properly assessed due to the small sample size. There was a significant difference in ASA score, with ASA class III being higher in the conventional care group, which can have an impact on the recovery and duration of the hospital stay. The study is open-labelled, so inherent bias is associated with it.

## Conclusions

A tailored approach to ERAS pathways in emergency settings, such as in our study on PPUD, demonstrated a significant postoperative recovery and shorter hospital stay in comparison to conventional care. With no major postoperative morbidity or mortality, t-ERAS appears to be a safe protocol to follow in an emergency. There was a significant difference in the ASA class, with a higher ASA in the conventional care group, which could have an impact on the results. The conventional care group had a longer duration of illness, though not significant enough to affect recovery post-operatively. Further, multicentre studies will be needed to formulate a definite ERAS protocol in emergency surgery settings.
